# *Rosmarinus officinalis* Essential Oil Improves Scopolamine-Induced Neurobehavioral Changes via Restoration of Cholinergic Function and Brain Antioxidant Status in Zebrafish (*Danio rerio*)

**DOI:** 10.3390/antiox9010062

**Published:** 2020-01-10

**Authors:** Luminita Capatina, Razvan Stefan Boiangiu, Gabriela Dumitru, Edoardo Marco Napoli, Giuseppe Ruberto, Lucian Hritcu, Elena Todirascu-Ciornea

**Affiliations:** 1 Department of Biology, Faculty of Biology, Alexandru Ioan Cuza University of Iasi, 700506 Iasi, Romania; capatina.luminita@yahoo.com (L.C.); boiangiu.razvan@yahoo.com (R.S.B.); gabriela.dumitru@uaic.ro (G.D.); ciornea@uaic.ro (E.T.-C.); 2 Institute of Biomolecular Chemistry, National Research Council ICB-CNR, 95126 Catania, Italy; edoardo.napoli@icb.cnr.it (E.M.N.); giuseppe.ruberto@icb.cnr.it (G.R.)

**Keywords:** *Rosmarinus officinalis*, essential oil, scopolamine, anxiety, memory, oxidative stress

## Abstract

*Rosmarinus officinalis* L. is a traditional herb with various therapeutic applications such as antibacterial, antioxidant, anti-inflammatory, antidepressant, and anticholinesterase activities, and can be used for the prevention or treatment of dementia. In the present study, we tested whether *Rosmarinus officinalis* L. could counteract scopolamine-induced anxiety, dementia, and brain oxidative stress in the zebrafish model and tried to find the underlying mechanism. *Rosmarinus officinalis* L. essential oil (REO: 25, 150, and 300 µL/L) was administered by immersion to zebrafish (*Danio rerio*) once daily for eight days while scopolamine (100 µM) treatment was delivered 30 min before behavioral tests. The antidepressant and cognitive-enhancing actions of the essential oil in the scopolamine zebrafish model was measured in the novel tank diving test (NTT) and Y-maze test. The chemical composition was identified by Gas chromatograph–Mass spectrometry (GC-MS) analysis. The brain oxidative status and acetylcholinesterase (AChE) activity was also determined. REO reversed scopolamine-induced anxiety, memory impairment, and brain oxidative stress. In addition, a reduced brain AChE activity following the administration of REO in scopolamine-treated fish was observed. In conclusion, REO exerted antidepressant-like effect and cognitive-enhancing action and was able to abolish AChE alteration and brain oxidative stress induced by scopolamine.

## 1. Introduction

Alzheimer’s disease (AD) is a progressive neurodegenerative disorder, particularly affecting the cerebral cortex and hippocampus, leading to memory impairment. AD pathological hallmarks include extracellular accumulation of insoluble forms of amyloid-β, aggregation of the microtubule protein tau in neurofibrillary tangles in neurons, as well as the reduction in levels of acetylcholine [1,2].

Among various descriptive hypotheses regarding the cause of AD, the cholinergic hypothesis was the first proposed to explain AD based on the findings that a loss of cholinergic activity is commonly observed in the brains of AD patients [1]. In addition, this theory implied utilizing acetylcholinesterase inhibitors (AChEIs), which reversed memory deficits in AD patients. AChEIs could diminish memory impairment in AD patients by inhibiting the degradation of acetylcholine [3]. Currently, for the treatment of mild to moderate AD, three AChEIs are used: donepezil, rivastigmine, and galantamine [4]. Moreover, it has been reported that the daily living ability of AD patients subjected to rivastigmine and galantamine medications is better than those treated with donepezil [5]. AD is additionally associated with neuropsychiatric symptoms such as depression, anxiety, and apathy [6]. Oxidative stress is involved in age-related diseases and the pathological processes of neurodegenerative diseases, including AD [7].

Presently, there is no remedy for dementia-related afflictions, and existing medicines do not give satisfactory enhancements. Moreover, there are different side effects related to existing treatment. Herbal-based compounds could be a great source of anti-AD agents [8].

Although numerous studies have been performed to elucidate the effects of *Rosmarinus officinalis* extract on cognitive function in rodents [9,10,11], there is no such study conducted in zebrafish. Zebrafish exhibit complex cognition comparable to that seen in mammals [12,13], and there are behavioral tasks protocols based on rodent protocols such as active or passive avoidance test [14], Y-maze test [15], and T-maze test [16] for zebrafish. In zebrafish, scopolamine, a muscarinic acetylcholine receptor blocker, has been characterized to induce amnestic effects and is used in combination with nootropic and cognitive-enhancing drugs to study memory processes [17].

Supporting data demonstrate different biological effects of *Rosmarinus officinalis* L. Nematolahi et al. [18] demonstrated that *Rosmarinus officinalis* reduced memory-deficits, anxiety, and depression, and improved sleep quality in university students. Naderali et al. [9] reported that *Rosmarinus officinalis* extract improved memory deficits and mitigated neuronal degeneration induced by kainic acid in the rat hippocampus, due to its antioxidant profile. In addition, Karim et al. [10], by molecular docking and in vivo approaches, demonstrated anti-amnesic effects of nepitrin isolated from *Rosmarinus officinalis* on scopolamine-induced memory impairment in mice. Song et al. [11] demonstrated that a rat model of repetitive mild traumatic brain injury subjected to *Rosmarinus officinalis* extract exhibited improvement of cognitive deficits mediated by its antioxidant and anti-inflammatory profile.

The biological activities of the *Rosmarinus officinalis* could be related to the presence of volatile compounds, such as α-pinene, eucalyptol, and camphor, and phenolic compounds, such as carnosol, carnosic acid, and rosmarinic acid, with proved antioxidant, antibacterial, antifungal, anti-inflammatory, anti-AD, antidepressant and anxiolytic effects [18,19]. Therefore, the present study was designed to characterize the chemical components of the *Rosmarinus officinalis* essential oil (REO) and to evaluate the effects on anxiety, memory performance, and brain antioxidant status in a scopolamine-induced a zebrafish model of amnesia.

## 2. Materials and Methods

### 2.1. Essential Oil and Chemical Material

REO from biological cultivations was manufactured and kindly supplied by Flora Srl (Pisa, Italy), batch number 171861. Pure standards of essential oil compounds (see Table 1) were purchased from Sigma-Aldrich Chemical Co. and Fluka Chemie.

### 2.2. Gas Chromatograph–Mass Spectrometry (GC-MS) Analysis

A Gas Chromatograph (Shimadzu GC-17A, Shimadzu, Milan, Italy) with Flame Ionization Detector (GC-FID) equipped with a 15 m × 0.1 mm × 0.1 mm capillary column (Supelco SPB^TM^-5, Merk KGaA, Darmstadt, Germany) was used. The operating condition followed those previously published [20]: 60 °C for 1 min, 60–280 °C at 10 °C/min, and then 280 °C for 1 min; injector temperature, 250 °C; and detector temperature, 280 °C with helium as carrier gas (1 mL/min). GC-MS analyses were performed with the same column and operative conditions mentioned above. Mass spectrometer parameters were the same as those previously published [21]. Component identification was based on comparison of their retention indexes with those relative to a mix of C_9_–C_22_ *n*-alkanes, computer matching of spectral MS data, and fragmentation patterns with those in libraries [22,23], and co-injections with authentic samples (see Table 1).

### 2.3. Animals

**Figure 1:**
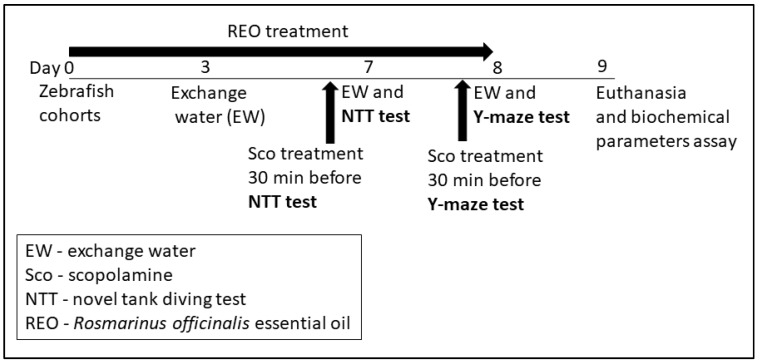
Scheme of the working protocol.

In total, 50 adult (sex ratio was about 50:50 male:female, 3–4-month old, and 3–4 cm in length), wild-type short-fin strain zebrafish (*Danio rerio*) purchased from an authorized commercial dealer (Pet Product S.R.L., Bucharest, Romania) were used in the present study and acclimated for at least two weeks before experiments. Fish were fed twice daily with Norwin Norvitall flake (Norwin, Gadstrup, Denmark). Animals were randomly divided into groups of 10 fish/24 L housing tanks filled with unchlorinated water under a 14 h/10 h light/dark cycle. The water within the tanks was constantly aerated (7.20 mg O_2_/L) using Tetra*tec*^®^ air pumps (Tetra, Melle, Germany) and filtrated to avoid the accumulation of organic toxins. The water parameters were kept in the following ranges: pH 7.5, conductivity 500 μS, ammonium concentration < 0.004 ppm, and temperature 26 ± 1 °C. For the behavior studies, acclimated zebrafish were randomly assigned into the control, the scopolamine (Sco, 100 µM, Sigma-Aldrich, Darmstadt, Germany), and three REO treatment groups (25, 150, and 300 µL/L). The doses of the essential oil were chosen with reference to previous reports [24]. REO (25, 150, and 300 µL/L) was administered individually by immersion to zebrafish (*Danio rerio*) through transferring into 500 mL glass for 1 h, once daily for eight days, whereas scopolamine (100 µM) treatment was delivered individually by transferring into a 500 mL glass, 30 min before behavioral tests. The control group was immersed only in unchlorinated water. The working protocol (as summarized in Figure 1) was approved by the local board of ethics for animal experimentation (No. 15309/2019) and fully complied with the Directive 2010/63/EU of the European Parliament and of the Council of 22 September 2010 on the protection of animals. Efforts were made to reduce animal suffering and the number of animals utilized.

### 2.4. Behavioral Analysis

#### 2.4.1. Novel Tank Diving Test (NTT)

NTT is a specific test used for assessing anxiety in zebrafish, as described by Cachat et al. [25]. The trapezoidal tank (1.5 L) used measured 15.2 cm (height) × 27.9 cm (top) × 22.5 cm (bottom) × 7.1 cm (width), equally divided into two horizontal sections (top and bottom). After 60 min of REO treatment, the animals were placed individually within the test tank without acclimatization, and swimming behavior was recorded for 6 min. The animals were recorded with a Logitech HD Webcam C922 Pro Stream camera (Logitech, Lausanne, Switzerland) placed 30 cm away from the tank and the videos were analyzed using ANY-Maze^®^ software (Stoelting CO, Wood Dale, IL, USA). The following parameters were registered: the number of entries in the top/bottom zone of the tank, total distance traveled in the tank (m), and average velocity (m/s).

#### 2.4.2. Y-Maze Test

Spatial memory and the response to novelty in zebrafish was assessed using the Y-maze task [26]. The position in the Y-maze task was considered an index of memory [27]. The apparatus consisted of a Y-maze glass aquarium with three arms (25 cm long, 8 cm wide, and 15 cm high), filled with 3 L of the same water used in the home aquarium. Visible geometric shapes such as squares, circles, and triangles were placed on the external Y-maze walls. The Y-maze arms were arbitrarily assigned: (i) the start arm, where fish began to investigate (always open); (ii) the novel arm, which was obstructed during the first trial, but open during the second trial; and (iii) the other arm (constantly open). The Y-maze center (neutral zone) was not counted in the analysis. The assignment comprised of two trials separated by 1 h to evaluate the response to novelty and spatial memory. During the first trial (training, 5 min), 60 min of REO treatment, the fish could investigate only two arms of the Y-maze (the start and the other arm), whereas the third arm (the novel arm) was obstructed. For the second trial, each fish was individually introduced into the start arm and free access to all three arms for 5 min to assess the response to novelty. Behavioral activity was analyzed using ANY-Maze^®^ software (Stoelting CO, Wood Dale, IL, USA) and with a Logitech HD Webcam C922 Pro Stream camera (Logitech, Lausanne, Switzerland) placed above the Y-maze tank. The following measures were recorded: time spent in each arm to assess short-term spatial memory and, for the locomotory activity total, distance traveled (m) and turn angle (°) were assessed.

### 2.5. Biochemical Parameters Assay

After the recording of behavioral data, zebrafish were euthanized (10 min immersion in ice water, 2–4 °C) until loss of opercular motions [28], and their brains were isolated for biochemical parameters assay. The brains were gently homogenized in ice 0.1 M potassium phosphate buffer (pH 7.4), 1.15% KCl with Potter Homogenizer (Cole-Parmer, Vernon Hills, IL, USA). The resulted homogenate was centrifuged at 960× *g* for 15 min. The supernatant was used for the estimation of acetylcholinesterase (AChE), superoxide dismutase (SOD), catalase (CAT), glutathione peroxidase (GPX) specific activities, and malondialdehyde (MDA) level following the methods described in detail by Dumitru et al. [29]. Estimation of protein content was done through a bicinchoninic acid (BCA) protein assay kit (Sigma-Aldrich, Darmstadt, Germany) [30].

### 2.6. Statistical Analysis

Data are expressed as mean ± standard error of the mean (S.E.M). Results were analyzed by one-way analysis of variance (ANOVA) followed by Tuckey’s post hoc multiple comparison test, considering treatment as a factor. Differences were considered significant at *p* < 0.05. GraphPad Prism 8.0 (GraphPad Software, Inc., San Diego, CA, USA) was used to perform statistical analyses and to produce graphics. Correlation among the behavioral scores, enzymatic activities, and lipid peroxidation was estimated by the Pearson correlation coefficient (*r*).

## 3. Results and Discussion

### 3.1. The Chemical Composition of the Essential Oil

**Figure 2:**
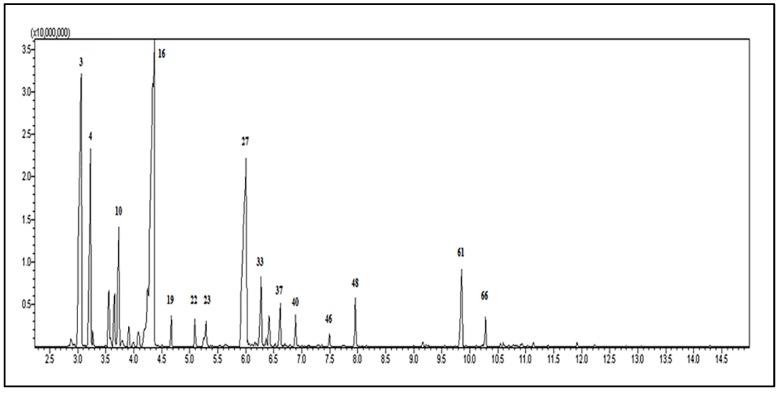
Gas chromatography-mass spectrometry (GC–MS) profile of the *Rosmarinus officinalis* essential oil (numbers refer to Table 1).

The chemical composition of the REO was identified by GC-MS analysis (Figure 2) and is presented in Table 1. Seventy-seven chemicals, corresponding to 73.48% of the total oil, were identified in the REO. The most abundant chemical classes of the oil components were monoterpene hydrocarbons (40.14%), followed by oxygenated monoterpenes (26.44%), sesquiterpene hydrocarbons (4.74%), and other compounds (2.16%). The major components of the REO were eucalyptol (26.02%), α-pinene (19.89%), camphor (16.71%), camphene (8.67%), β-myrcene (3.97%), β-caryophyllene (3.11%), borneol (2.50%), and limonene (2.16%). Our results are supported by Bouyahya et al. [31], who reported eucalyptol (23.673%), camphor (18.743%), borneol (15.46%), and α-pinene (14.076%) as the major chemical components of the REO. In addition, Elyemni et al. [32] reported the presence of eucalyptol (32.18%), camphor (16.20%), α-pinene (15.82%), camphene (9.16%), and α-terpineol (7.36%) in the chemical composition of the REO. Based on these studies, our essential oil exhibits a chemical composition commensurate to those reported by other authors who presume its memory-enhancing and antioxidant activity.

### 3.2. Effects on Anxiety-Like Behavior in NTT Test and on Y-Maze Response to Novelty and Spatial Memory

**Figure 3:**
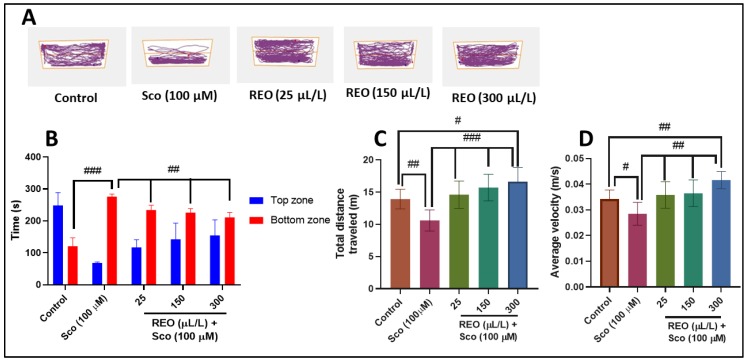
*Rosmarinus officinalis* essential oil (REO: 25, 150, and 300 µL/L) improved locomotion pattern and decreased anxiety in the NTT test. (**A**) Representative locomotion tracking pattern of the control, Sco (100 µM), and REO (25, 150, and 300 µL/L) treated groups. (**B**) The time spent by zebrafish in the top/bottom zone of the tank in different groups. (**C**) The total distance traveled by zebrafish in the tank in different groups. (**D**) The average velocity of zebrafish in the tank in different groups. Values are means ± S.E.M. (*n* = 10). For Tukey’s post hoc analyses: (**B**) Control vs. Sco (100 µM): ### *p* < 0.0001, Sco vs. REO (25 µL/L): ## *p* < 0.001, Sco vs. REO (150 µL/L): ## *p* < 0.001, and Sco vs. REO (300 µL/L): ## *p* < 0.001; (**C**) Control vs. Sco (100 µM): ### *p* < 0.0001, Control vs. REO (300 µL/L): # *p* < 0.01, Sco vs. REO (25 µL/L): ### *p* < 0.0001, Sco vs. REO (150 µL/L): ### *p* < 0.0001, and Sco vs. REO (300 µL/L): ### *p* < 0.0001; and (**D**) Control vs. Sco (100 µM): # *p* < 0.01, Control vs. REO (300 µL/L): ## *p* < 0.001, Sco vs. REO (25 µL/L): ### *p* < 0.0001, Sco vs. REO (150 µL/L): ### *p* < 0.0001, and Sco vs. REO (300 µL/L): ### *p* < 0.0001.

Figure 3 reports the effects of Sco (100 µM) and REO (25, 150, and 300 µL/L) treatment on anxiety-like behavior in the NTT test. Representative locomotion tracking pattern (Figure 3A) illustrates the differences in swimming traces among the top and bottom zones and shows that the Sco-treated group traveled a greater distance in the bottom zone, suggesting an anxiogenic profile. Additionally, Sco treatment increased the time spent in the bottom zone of the tank (*p* < 0.0001) (Figure 3B) along with decreasing the time spent in the top zone of the tank (Figure 3B) (*p* < 0.0001) as compared to control. Reducing the time spent in the top zone of the tank suggests the anxiogenic-like effect of Sco. Sco treatment produced a hypolocomotor effect, by decreasing total distance traveled (*p* < 0.001) (Figure 3C) and average velocity (i.e., magnitude and direction of zebrafish speed, *p* < 0.01) (Figure 3D) compared to control. By contrast, increasing the time spent in the top zone of the tank (Figure 3B) suggests the anxiolytic-like effect of REO. Moreover, treatment with REO prevents the anxiogenic-like effect of Sco, in a dose-dependent manner, as evidenced through increasing of total distance traveled (*p* < 0.0001) (Figure 3C) and average velocity (*p* < 0.001) (Figure 3D) as compared to Sco-alone treated fish.

**Figure 4:**
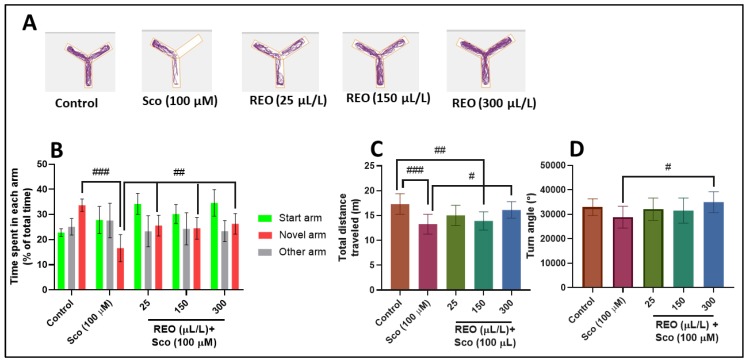
*Rosmarinus officinalis* essential oil (REO: 25, 150, and 300 µL/L) improved locomotion pattern and memory in the Y-maze test. (**A**) Representative locomotion tracking of the control, Sco (100 µM), and REO (25, 150, and 300 µL/L) treated groups. (**B**) The time spent in each arm (start, novel, and novel arms) in different groups. (**C**) The total distance traveled by zebrafish in the tank in different groups. (**D**) The turn angle of zebrafish in the tank in different groups. Values are means ± S.E.M. (*n* = 10). For Tukey’s post hoc analyses: (**B**) Control vs. Sco (100 µM): ### *p* < 0.0001, Sco vs. REO (25 µL/L): ## *p* < 0.001, Sco vs. REO (150 µL/L): ## *p* < 0.001, and Sco vs. REO (300 µL/L): ## *p* < 0.001; (**C**) Control vs. Sco (100 µM): ### *p* < 0.0001, Control vs. Sco (100 µM): ## *p* < 0.001, and Sco vs. REO (300 µL/L): # *p* < 0.01; and (**D**) Sco vs. REO (300 µL/L): # *p* < 0.01.

Figure 4 shows the effects of Sco (100 µM) and REO (25, 150, and 300 µL/L) treatment on Y-maze response to novelty and spatial memory. Representative locomotion tracking pattern (Figure 4A) illustrates the differences in swimming traces among the Y-maze arms and shows that Sco treated group traveled a greater distance in the other arm, suggesting memory deficits. In addition, Sco administration significantly altered novel arm exploration (*p* < 0.0001) (Figure 4B) as compared to control zebrafish. The reduced percentage of time spent in novel arm suggests a memory impairment effect of Sco. The administration of Sco affects locomotion, as evidenced by the decreasing of total distance traveled (*p* < 0.0001) (Figure 4C) and turn angle (*p* > 0.05) (Figure 4D) compared to control. REO treatment significantly counters the Sco action induced hypolocomotion and memory deficits by improving the novel arm exploration (*p* < 0.001) (Figure 4B), while total distance traveled (*p* < 0.01) (Figure 4C) and turn angle (*p* < 0.01) (Figure 4D) was significantly increased at the high doses of REO (300 µL/L) as compared to Sco-alone treated zebrafish. Our results demonstrate that REO exhibited anxiolytic and memory-enhancing profile, which could be due to the presence of major active constituents shown in Table 1. The obtained results are in perfect agreement with those obtained by other groups that demonstrated anti-amnesic effects along with in vitro antioxidant and acetylcholinesterase and butyrylcholinesterase inhibition potential of *Rosmarinus officinalis* in scopolamine-induced memory impairment in mice [10]. Ozarowski et al. [33] demonstrated that *Rosmarinus officinalis* leaf extract improved long-term memory in rats, which can be partially explained by its inhibition of AChE activity in rat brain. Noori Ahmad Abadi et al. [34] reported that the hydroalcoholic extract of *Rosmarinus officinalis* L. leaf reduced anxiety in mice, probably due to the presence of flavonoids in this plant and their antioxidant property. Additionally, Abdelhalim et al. [35] attributed the anxiolytic effects of *Rosmarinus officinalis* with its effect on gamma-aminobutyric acid (GABA) receptors. Despite extensive knowledge about the effects of various *Rosmarinus officinalis* extracts on memory, anxiety, and AChE activity in the rodent brain, we demonstrated for the first time the cognitive-enhancing, anxiolytic, and antioxidant profile of REO in the scopolamine zebrafish model. Furthermore, our study demonstrated that zebrafish is rapidly becoming one of the main organisms in translational neuroscience research, successfully completing rodent models for the study of dementia-related conditions.

### 3.3. Effects on AChE Activity

**Figure 5:**
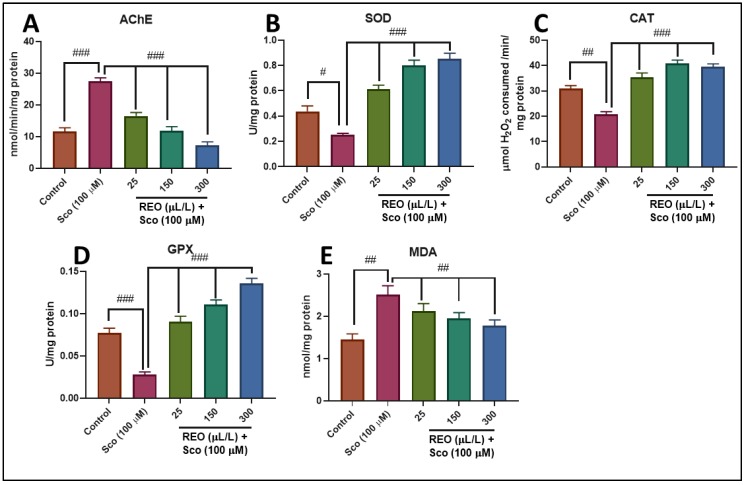
*Rosmarinus officinalis* essential oil (REO: 25, 150, and 300 µL/L) exhibited an anti-AChE effect and improved brain antioxidant status. The enzyme’s specific activities: (**A**) AChE; (**B**) SOD; (**C**) CAT; (**D**) GPX; and (**E**) MDA level. Values are means ± S.E.M. (*n* = 10). For Tukey’s post hoc analyses: (**A**) Control vs. Sco (100 µM): ### *p* < 0.0001, Sco vs. REO (25 µL/L): ### *p* < 0.0001, Sco vs. REO (150 µL/L): ### *p* < 0.0001, and Sco vs. REO (300 µL/L): ### *p* < 0.0001; (**B**) Control vs. Sco (100 µM): # *p* < 0.01, Sco vs. REO (25 µL/L): ### *p* < 0.0001, Sco vs. REO (150 µL/L): ### *p* < 0.0001, and Sco vs. REO (300 µL/L): ### *p* < 0.0001; (**C**) Control vs. Sco (100 µM): ## *p* < 0.001, Sco vs. REO (25 µL/L): ### *p* < 0.0001, Sco vs. REO (150 µL/L): ### *p* < 0.0001, and Sco vs. REO (300 µL/L): ### *p* < 0.0001; (**D**) Control vs. Sco (100 µM): ### *p* < 0.0001, Sco vs. REO (25 µL/L): ### *p* < 0.0001, Sco vs. REO (150 µL/L): ### *p* < 0.0001, and Sco vs. REO (300 µL/L): ### *p* < 0.0001; and (**E**) Control vs. Sco (100 µM): ## *p* < 0.001, Sco vs. REO (25 µL/L): ### *p* < 0.0001, Sco vs. REO (150 µL/L): ### *p* < 0.0001, and Sco vs. REO (300 µL/L): ### *p* < 0.0001.

AChE decreases acetylcholine level and alleviates disease symptoms associated with the progressive loss of cholinergic function in AD [36]. Our results demonstrate that zebrafish subjected to Sco treatment exhibited increased AChE activity in the brain (*p* < 0.0001) as compared to the control group (Figure 5A). REO treatment significantly reduced AChE activity (*p* < 0.0001) in a dose-dependent manner, as compared to the Sco-treated group (Figure 5A). Thus, REO revealed an anti-AChE profile [19], which parallels improving memory parameters in zebrafish, as observed in NTT and Y-maze tests.

### 3.4. Effects on SOD, CAT, and GPX Specific Activities

Administration of Sco decreased the SOD specific activity (*p* < 0.01) (Figure 5B) in the zebrafish brain as compared to the control group, suggesting the augmentation in oxidative stress. REO significantly increase (*p* < 0.0001) (Figure 5B) the specific activity of SOD in the Sco-treated fish showing its promising antioxidant potential. CAT specific activity significantly decreased (*p* < 0.01) (Figure 5C) in Sco-exposed zebrafish as compared to the control group, whereas the administration of REO led to a significant increase (*p* < 0.0001) (Figure 5C) of CAT activity in the Sco-treated fish, supporting its antioxidant action. Moreover, GPX specific activity upon Sco administration leads to a significant decrease (*p* < 0.0001) (Figure 5D) as compared to the control group. REO treatment significantly restored this dramatic decrease in the Sco-treated fish showing prompt antioxidant potential. The results suggest that REO exhibits neuroprotective effects against oxidative stress, which is correlated with previously antioxidant properties of REO. Selmi et al. [37] reported that REO administration has significantly protected against alloxan-induced hepatic and renal oxidative stress due to the presence of phenolic and flavonoids compounds. Takayama et al. [38] demonstrated the antioxidant activity of REO against gastric damage induced by absolute ethanol in the rat. El-Hadary et al. [39] demonstrated antioxidant properties of REO against carbon tetrachloride-induced hepatotoxicity in rats mediated by phenolic compounds. Our results in accordance with the literature, suggesting the ability of REO to control brain oxidative damages by restoring the antioxidant enzyme activities.

### 3.5. Effects on MDA Level

The MDA level, an indicator of lipid peroxidation, was increased (*p* < 0.001) (Figure 5E) in the Sco-treated fish as compared to the control group. REO administration significantly reduced the MDA level (*p* < 0.001) (Figure 5E) to near the control level in Sco-treated fish.

**Figure 6:**
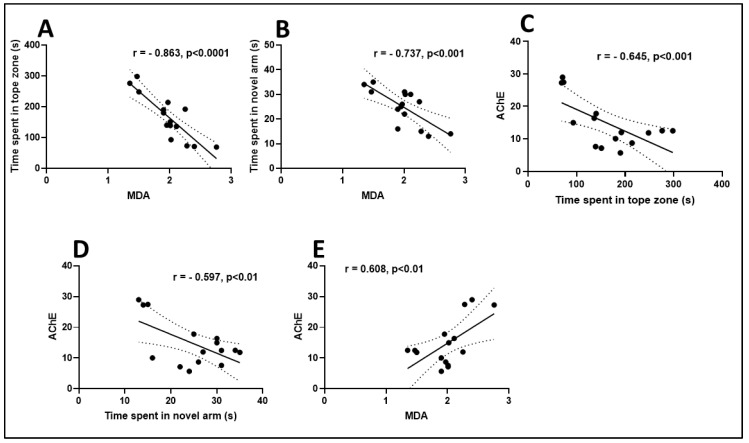
Correlation analyses between behavioral and biochemical parameters (Pearson’s correlation, *n* = 10): (**A**) time spent in tope zone (s) vs. MDA; (**B**) time spent in novel arm (s) vs. MDA; (**C**) AChE vs. time spent in tope zone (s); (**D**) AChE vs. time spent in novel arm (s) and (**E**) AChE vs. MDA. Data expressed are time spent in the top zone (s), time spent in novel arm (s), AChE (nmol/min/mg protein), and MDA (nmol/mg protein).

Pearson correlation coefficient (*r*) was used to test the linear association among cognition, antioxidant enzymes, and lipid peroxidation (Figure 6). A high negative correlation for the time spent in the top zone of the tank vs. MDA (*n* = 10, *r* = −0.863, *p* < 0.001) (Figure 6A) and the time spent in novel arm vs. MDA (*n* = 10, *r* = −0.737, *p* < 0.001) (Figure 6B) was observed. The negative value of the Pearson correlation coefficient indicates that the improvement of behavioral scores in specific tests such as NTT and Y-maze is well correlated with a low level of MDA, a marker of lipid peroxidation. In addition, strong negative correlations were evidenced by linear regression for AChE vs. the time spent in the top zone of the tank (*n* = 10, *r* = −0.645, *p* < 0.001) (Figure 6C) and AChE vs. the time spent in novel arm (*n* = 10, *r* = −0.597, *p* < 0.01) (Figure 6D). However, a positive significant correlation for AChE vs. MDA (*n* = 10, *r* = 0.608, *p* < 0.01) (Figure 6E) was noticed when linear regression was calculated. In this case, the negative and positive values of the Pearson correlation coefficient indicate that increasing behavioral scores is well correlated with decreasing of AChE activity and MDA level. Pérez-Fons et al. [40] demonstrated a relationship between the antioxidant capacity and effect of rosemary (*Rosmarinus officinalis* L.) polyphenols on membrane phospholipid order. By using the Pearson correlation coefficient (*r*) determination, we evidenced that improving memory performance in Sco-treated rats is related to increasing antioxidant enzyme activity along with a diminished level of lipid peroxidation, supporting REO neuroprotective profile. No significant correlation between biochemical parameters was observed.

## 4. Conclusions

This study showed that REO counteracts cognitive performance decrease and anxiety increase resulting from Sco treatment through a mechanism implying mitigation of brain oxidative stress and regulation of AChE activity.

## Figures and Tables

**Table 1:** Chemical composition of *Rosmarinus officinalis* essential oil ^a^.

No.	KI ^b^	Compound	%
		**Monoterpene hydrocarbons**	**40.14**
1	920	Tricyclene	0.32
2	925	α-Thujene	0.09
3	934	α-Pinene ^c^	19.89
4	949	Camphene	8.67
5	953	Thuja-2.4(10)-diene	0.33
6	972	Sabinene	0.02
7	975	β-Pinene ^c^	1.56
10	988	β-Myrcene	3.97
12	1001	α-Phellandrene	0.63
13	1008	α-Terpinene	0.15
14	1015	*p*-Cymene	0.68
15	1024	Limonene ^c^	2.16
17	1037	*cis*-β-Ocimene	0.16
18	1047	*trans*-β-Ocimene	0.04
19	1058	γ-Terpinene	0.74
22	1087	Terpinolene	0.75
		**Oxygenated monoterpenes**	**26.44**
16	1033	Eucalyptol ^c^	26.02
20	1070	*cis*-Sabinene hydrate	0.01
21	1075	*cis*-Linalool oxide	0.01
23	1098	Linalool	1.10
24	1109	*endo*-Fenchol	0.02
25	1115	*exo*-Fenchol	0.06
26	1125	α-Campholenal	0.14
27	1148	Camphor ^c^	16.71
28	1151	Camphene hydrate	0.04
29	1155	Menthone	0.03
30	1158	Isopulegol	0.03
31	1162	*trans*-Pinocamphone	0.07
32	1164	Pinocarvone	0.07
33	1168	Borneol	2.50
34	1175	*cis*-Pinocamphone	0.17
35	1178	Terpinen-4-ol	0.78
36	1186	*p*-Cymen-8-ol	0.08
37	1191	*a*-Terpineol	1.38
38	1198	Myrtenol	0.10
39	1204	*trans*-Dihydro Carvone	0.06
40	1210	Verbenone	1.18
41	1221	*trans*-Carveol	0.01
42	1231	Linalyl formate	0.06
43	1242	*cis*-Dihydro Carvone	0.03
44	1244	Neral	0.01
45	1248	Carvone	0.03
46	1258	Linalyl acetate	0.35
47	1275	Geranial	0.01
48	1289	Bornyl acetate	1.10
49	1299	Thymol	0.01
50	1305	Carvacrol	0.03
51	1327	Piperitenone	0.02
52	1348	Eugenol	0.01
53	1361	Neryl acetate	0.01
54	1366	Linalyl isobutanoate	0.02
55	1376	α-ylangene	0.11
56	1380	α-Copaene	0.06
57	1384	Geranyl acetate	0.03
58	1403	Methyl eugenol	0.02
		**Sesquiterpenes**	**4.74**
59	1410	α-Caryophyllene	0.01
60	1418	α-*cis*-Bergamotene	0.03
61	1425	β-Caryophyllene ^c^	3.11
62	1433	β-Ylangene	0.02
63	1444	β-Bergamotene	0.01
64	1447	β-Copaene	0.02
65	1454	Aromadendrene	0.04
66	1459	α-Humulene	0.88
67	1480	γ-Muurolene	0.07
68	1484	α-Curcumene	0.09
69	1491	β-Selinene	0.02
70	1497	γ-Amorphene	0.06
71	1508	α-Muurolene	0.02
72	1510	β-Bisabolene	0.05
73	1518	γ-Cadinene	0.05
74	1527	δ-Cadinene	0.12
75	1543	α-Cadinene	0.01
76	1548	α-Calacorene	0.04
77	1589	Caryophyllene oxide	0.10
		**Others**	**2.16**
8	977	Octen-3-ol	0.37
9	982	3-Octanone	1.56
11	993	3-Octanol	0.23

^a^ The numbering refers to the elution order, and values (relative peak area percent) represent averages of three determinations; ^b^ Retention Index (KI) relative to a standard mixture of *n*-alkanes on the SPB^TM^-5 column; ^c^ co-elution with an authentic sample.

## References

[1] Craig L.A., Hong N.S., Kopp J., McDonald R.J. (2008). Reduced cholinergic status in hippocampus produces spatial memory deficits when combined with kainic acid induced seizure. Hippocampus.

[2] Masters C.L., Bateman R., Blennow K., Rowe C.C., Sperling R.A., Cummings J.L. (2015). Alzheimer’s disease. Nat. Rev. Dis. Prim..

[3] Liu P.-P., Xie Y., Meng X.-Y., Kang J.-S. (2019). History and progress of hypotheses and clinical trials for Alzheimer’s disease. Signal Transduct. Target. Ther..

[4] Doody R.S., Dunn J.K., Clark C.M., Farlow M., Foster N.L., Liao T., Gonzales N., Lai E., Massman P. (2001). Chronic Donepezil Treatment Is Associated with Slowed Cognitive Decline in Alzheimer’s Disease. Dement. Geriatr. Cogn. Disord..

[5] Bullock R., Touchon J., Bergman H., Gambina G., He Y., Rapatz G., Nagel J., Lane R. (2005). Rivastigmine and donepezil treatment in moderate to moderately-severe Alzheimer’s disease over a 2-year period. Curr. Med. Res. Opin..

[6] Lyketsos C.G., Carrillo M.C., Ryan J.M., Khachaturian A.S., Trzepacz P., Amatniek J., Cedarbaum J., Brashear R., Miller D.S. (2011). Neuropsychiatric symptoms in Alzheimer’s disease. Alzheimer’s Dement..

[7] Tönnies E., Trushina E. (2017). Oxidative Stress, Synaptic Dysfunction, and Alzheimer’s Disease. J. Alzheimer’s Dis..

[8] Jafarian S., Ling K.-H., Hassan Z., Perimal-Lewis L., Sulaiman M.R., Perimal E.K. (2019). Effect of zerumbone on scopolamine-induced memory impairment and anxiety-like behaviours in rats. Alzheimer’s Dement..

[9] Naderali E., Nikbakht F., Ofogh S., Rasoolijazi H. (2018). The role of rosemary extract (40% carnosic acid) in degeneration of hippocampal neurons induced by kainic acid in the rat: The behavioral and histochemical approach. J. Integr. Neurosci..

[10] Karim N., Khan I., Abdelhalim A., Abdel-Halim H., Hanrahan J.R. (2017). Molecular docking and antiamnesic effects of nepitrin isolated from *Rosmarinus officinalis* on scopolamine-induced memory impairment in mice. Biomed. Pharmacother..

[11] Song H., Xu L., Zhang R., Cao Z., Zhang H., Yang L., Guo Z., Qu Y., Yu J. (2016). Rosemary extract improves cognitive deficits in a rats model of repetitive mild traumatic brain injury associated with reduction of astrocytosis and neuronal degeneration in hippocampus. Neurosci. Lett..

[12] Gerlai R. (2017). Zebrafish and relational memory: Could a simple fish be useful for the analysis of biological mechanisms of complex vertebrate learning?. Behav. Process..

[13] Meshalkina D.A., Kizlyk M.N., Kysil E.V., Collier A.D., Echevarria D.J., Abreu M.S., Barcellos L.J.G., Song C., Kalueff A.V. (2017). Understanding zebrafish cognition. Behav. Process..

[14] Xu X., Scott-Scheiern T., Kempker L., Simons K. (2007). Active avoidance conditioning in zebrafish (Danio rerio). Neurobiol. Learn. Mem..

[15] Aoki R., Tsuboi T., Okamoto H. (2015). Y-maze avoidance: An automated and rapid associative learning paradigm in zebrafish. Neurosci. Res..

[16] Braida D., Ponzoni L., Martucci R., Sparatore F., Gotti C., Sala M. (2014). Role of neuronal nicotinic acetylcholine receptors (nAChRs) on learning and memory in zebrafish. Psychopharmacology.

[17] Hamilton T.J., Morrill A., Lucas K., Gallup J., Harris M., Healey M., Pitman T., Schalomon M., Digweed S., Tresguerres M. (2017). Establishing zebrafish as a model to study the anxiolytic effects of scopolamine. Sci. Rep..

[18] Nematolahi P., Mehrabani M., Karami-Mohajeri S., Dabaghzadeh F. (2018). Effects of *Rosmarinus officinalis* L. on memory performance, anxiety, depression, and sleep quality in university students: A randomized clinical trial. Complement. Ther. Clin. Pract..

[19] Miraj S. (2016). An evidence-based review on herbal remedies of *Rosmarinus officinalis*. Der Pharm. Lett..

[20] Napoli E.M., Curcuruto G., Ruberto G. (2010). Screening of the essential oil composition of wild Sicilian rosemary. Biochem. Syst. Ecol..

[21] Tuttolomondo T., Dugo G., Ruberto G., Leto C., Napoli E.M., Potortí A.G., Fede M.R., Virga G., Leone R., D’Anna E. (2015). Agronomical evaluation of Sicilian biotypes of Lavandula stoechas L. spp. stoechas and analysis of the essential oils. J. Essent. Oil Res..

[22] Epa N., Mass N.I.H., Library S., Ei V.Z., Sparkman J.A. (2004). NIST Standard Reference Database 1A. Natl. Inst. Stand. Technol. NIST.

[23] Sparkman O.D. (2007). Review. J. Am. Soc. Mass Spectrom..

[24] Dos Santos A.C., Junior G.B., Zago D.C., Zeppenfeld C.C., da Silva D.T., Heinzmann B.M., Baldisserotto B., da Cunha M.A. (2017). Anesthesia and anesthetic action mechanism of essential oils of Aloysia triphylla and Cymbopogon flexuosus in silver catfish (Rhamdia quelen). Vet. Anaesth. Analg..

[25] Cachat J.M., Canavello P.R., Elkhayat S.I., Bartels B.K., Hart P.C., Elegante M.F., Beeson E.C., Laffoon A.L., Haymore W.A.M., Tien D.H. (2011). Video-aided analysis of zebrafish locomotion and anxiety-related behavioral responses. Neuromethods.

[26] Cognato G.d.P., Bortolotto J.W., Blazina A.R., Christoff R.R., Lara D.R., Vianna M.R., Bonan C.D. (2012). Y-Maze memory task in zebrafish (Danio rerio): The role of glutamatergic and cholinergic systems on the acquisition and consolidation periods. Neurobiol. Learn. Mem..

[27] Zanandrea R., Abreu M.S., Piato A., Barcellos L.J.G., Giacomini A.C.V.V. (2018). Lithium prevents scopolamine-induced memory impairment in zebrafish. Neurosci. Lett..

[28] Batista F.L.A., Lima L.M.G., Abrante I.A., de Araújo J.I.F., Batista F.L.A., Abrante I.A., Magalhães E.A., de Lima D.R., Lima M.d.C.L., do Prado B.S. (2018). Antinociceptive activity of ethanolic extract of Azadirachta indica A. Juss (Neem, Meliaceae) fruit through opioid, glutamatergic and acid-sensitive ion pathways in adult zebrafish (Danio rerio). Biomed. Pharmacother..

[29] Dumitru G., El-Nashar H.A.S., Mostafa N.M., Eldahshan O.A., Boiangiu R.S., Todirascu-Ciornea E., Hritcu L., Singab A.N.B. (2019). Agathisflavone isolated from Schinus polygamus (Cav.) Cabrera leaves prevents scopolamine-induced memory impairment and brain oxidative stress in zebrafish (Danio rerio). Phytomedicine.

[30] Smith P.K., Krohn R.I., Hermanson G.T., Mallia A.K., Gartner F.H., Provenzano M.D., Fujimoto E.K., Goeke N.M., Olson B.J., Klenk D.C. (1985). Measurement of protein using bicinchoninic acid. Anal. Biochem..

[31] Bouyahya A., Et-Touys A., Bakri Y., Talbaui A., Fellah H., Abrini J., Dakka N. (2017). Chemical composition of *Mentha pulegium* and *Rosmarinus officinalis* essential oils and their antileishmanial, antibacterial and antioxidant activities. Microb. Pathog..

[32] Elyemni M., Louaste B., Nechad I., Elkamli T., Bouia A., Taleb M., Chaouch M., Eloutassi N. (2019). Extraction of Essential Oils of *Rosmarinus officinalis* L. by Two Different Methods: Hydrodistillation and Microwave Assisted Hydrodistillation. Sci. World J..

[33] Ozarowski M., Mikolajczak P.L., Bogacz A., Gryszczynska A., Kujawska M., Jodynis-Liebert J., Piasecka A., Napieczynska H., Szulc M., Kujawski R. (2013). *Rosmarinus officinalis* L. leaf extract improves memory impairment and affects acetylcholinesterase and butyrylcholinesterase activities in rat brain. Fitoterapia.

[34] Noori Ahmad Abadi M., Mortazavi M., Kalani N., Marzouni H.Z., Kooti W., Ali-Akbari S. (2016). Effect of Hydroalcoholic Extract of *Rosmarinus officinalis* L. Leaf on Anxiety in Mice. J. Evid. Based Complement. Altern. Med..

[35] Abdelhalim A., Karim N., Chebib M., Aburjai T., Khan I., Johnston G.A.R., Hanrahan J.R. (2015). Antidepressant, anxiolytic and antinociceptive activities of constituents from *Rosmarinus officinalis*. J. Pharm. Pharm. Sci..

[36] Li S.M., Mo M.S., Xu P.Y. (2015). Progress in mechanisms of acetylcholinesterase inhibitors and memantine for the treatment of Alzheimer’s disease. Neuroimmunol. Neuroinflamm..

[37] Selmi S., Rtibi K., Grami D., Sebai H., Marzouki L. (2017). Rosemary (*Rosmarinus officinalis*) essential oil components exhibit anti-hyperglycemic, anti-hyperlipidemic and antioxidant effects in experimental diabetes. Pathophysiology.

[38] Takayama C., de-Faria F.M., de Almeida A.C.A., Dunder R.J., Manzo L.P., Socca E.A.R., Batista L.M., Salvador M.J., Souza-Brito A.R.M., Luiz-Ferreira A. (2016). Chemical composition of *Rosmarinus officinalis* essential oil and antioxidant action against gastric damage induced by absolute ethanol in the rat. Asian Pac. J. Trop. Biomed..

[39] El-Hadary A.E., Elsanhoty R.M., Ramadan M.F. (2019). In vivo protective effect of *Rosmarinus officinalis* oil against carbon tetrachloride (CCl4)-induced hepatotoxicity in rats. PharmaNutrition.

[40] Pérez-Fons L., GarzÓn M.T., Micol V. (2010). Relationship between the Antioxidant Capacity and Effect of Rosemary (*Rosmarinus officinalis* L.) Polyphenols on Membrane Phospholipid Order. J. Agric. Food Chem..

